# Sedentary Lifestyle and Nonspecific Low Back Pain in Medical Personnel in North-East Poland

**DOI:** 10.1155/2018/1965807

**Published:** 2018-09-09

**Authors:** Anna Citko, Stanisław Górski, Ludmiła Marcinowicz, Anna Górska

**Affiliations:** ^1^Department of Family Medicine, Medical University of Bialystok, Poland; ^2^Department of Medical Education, Jagiellonian University Medical College, Cracow, Poland; ^3^Department of Primary Health Care, Medical University of Bialystok, Poland

## Abstract

**Introduction:**

The sedentary lifestyle is defined as prolonged sitting both at work and during leisure time, with energy expenditures of below 600 MET · min/week. The sedentary lifestyle is a well-known predictor of obesity and other components of the metabolic syndrome. The influence of the sedentary lifestyle and associated factors on nsLBP is still being discussed.

**Aim:**

The aim of this study was to assess the influence of a sedentary lifestyle and its associated metabolic predictors on the prevalence of nsLBP in nurses and paramedics.

**Materials and Methods:**

The study included 609 participants, aged 30-60 years, who were residents of north-east Poland. Data was collected using a questionnaire (based, in part, on the Nordic Musculoskeletal Questionnaire), and included details of sociodemographic profile, chronic illnesses, and a short version of the International Physical Activity Questionnaire (IPAQ).

**Results:**

Nearly half (49.59%) of the respondents reported decreased physical activity, and in the group with recurring nsLBP this figure was 67.59%. Univariate logistic regression modelling found that leading a sedentary lifestyle caused a 3.5-fold increase in the incidence of recurring nsLBP (p<0.001). Excessive coffee consumption significantly increased the likelihood of recurring LBP (OR=16.44, 95% CI: 8.55-31.61), and cigarette smoking increased the likelihood of both recurrent and chronic LBP. The likelihood of chronic low back pain was significantly increased by components of metabolic syndrome such as high blood pressure (over 9-fold), type 2 diabetes (over 3-fold), and hyperlipidemia (over 2-fold) (p<0.001, p<0.001, and p<0.01, respectively).

**Conclusions:**

A sedentary lifestyle significantly increased the incidence of recurring low back pain, while increased physical activity had a significant effect on the presence of chronic low back pain. In the sedentary lifestyle group, conditions classified within metabolic syndrome were found to significantly increase the chances of developing nonspecific low back pain.

## 1. Introduction

Nonspecific low back pain (nsLBP) is a mounting health problem in the 21^st^ century. After headaches, it is the second most common pain reported [1]. NsLBP was ranked in first place for the most common reasons for disability on the YLD (Years Lived with Disability) metric. In recent reports, the increased incidence of nsLBP in young and middle-aged persons is emphasized [2]. The diagnosis of nsLBP is given when no other cause for back pain, apart from spinal degeneration, can be established.

It is suggested that the sedentary lifestyle is one of the predictors for nsLBP [3]. The sedentary lifestyle is defined as prolonged sitting at work, during leisure time and when moving; these activities require energy expenditures of <1.5 METS. An average weekly energy expenditure expressed as MET·min/week for efforts associated with different types of activities (professional work, moving, house chores, and recreation and tourism) does not exceed 600 MET·min/week [4]. Lack of or trace physical activity resulting from the sedentary lifestyle results in the reduction of muscular power and strength [3]. Furthermore, the sedentary lifestyle leads to a reduced ability of the vertebral disc to maintain a normal concentration of water. The level of hydration of the nucleus pulposus influences development of degenerative and overload lesions. It was also found that the sedentary lifestyle may be a risk factor for vertebral disc herniation [5]. People with a sedentary lifestyle may develop flaccid hyperlordosis complex resulting in the development of nsLBP.

Nurses and paramedics are a group of professionals who are at a high risk of developing pain in the lumbar section of the spine. Working with patients often involves excessive strain on the low back resulting from the need to maintain a forced body position (referred to as postural stress). In finding effective preventative approaches for nsLBP, it seems reasonable to study factors associated not only with working environment, but also lifestyle factors such as daily physical activity, cigarette smoking, excessive coffee consumption, and poor diet. There is a reported association between a lifestyle and the incidence of so-called diseases of affluence. [6, 7].

The importance of overweight or obesity as risk factors for low back pain is also noted [8, 9]. Both overweight and obesity contribute to the mechanical overload of paraspinal tissues or even promotes the development of disc herniations. There have been reports on the influence of other metabolic disorders (hyperlipidaemia, hypertension, and type 2 diabetes) on the occurrence of low back pain [10, 11]. Less numerous reports indicate a relationship between smoking and low back pain [11].

As an increase in low back pain frequency is observed, a research project bringing the knowledge pertaining to the link between sedentary lifestyle and the occurrence of nsLBP appears to be justified.

The aim of this study was to assess the influence of a sedentary lifestyle and its associated metabolic predictors on the prevalence of nonspecific low back in nurses and paramedics.

## 2. Methods

A group of 609 randomly selected people (medical staff) participated in the study. They lived in north-eastern Poland and their age ranged from 30 to 60 years (the average age was 40.99 ± 6.66 years). In total, 324 nurses (53.2%) and 285 paramedics (46.8%) were included in the study. Among the subjects, there were 302 people aged 30–40 (49.6%) and 307 people aged 41–60 (50.4%). As far as the sex of the participants was concerned, women constituted 59.44% (362 people) and men 40.56% (247 people). As far as the value of the body mass index (BMI) was concerned, it was established that 314 (51.56%) people had correct body mass (BMI = 18.5–24.99 kg/m^2^) while 295 (48.44%) subjects were overweight (BMI = 25–29.99 kg/m^2^) or obese (BMI ≥ 30 kg/m^2^). Detailed characteristics of the study group are shown in Table 1.

The research was carried out using an auditorial survey supervised by researchers, i.e., the authors of the manuscript.

The following types of questionnaires were used:

(1) The questionnaire partially based on standardized Nordic Musculoskeletal Questionnaire includes questions regarding frequency of musculoskeletal pain (1-2 times within past 12 months, ≥ 3 times within 12 months; the pain is present since at least 12 weeks) within low back.

An acute pain episode which had occurred ≥ 3 times in the last 12 months was considered “recurring” low back pain [1, 12]. Chronic back pain is defined as pain that persists for 12 weeks or longer [1].

(2) Author's questionnaire regarding sociodemographic details selected recurrent and chronic diseases (hyperlipidemia, arterial hypertension, and diabetes type 2). This questionnaire was composed of closed questions to choose from (disjunctive question). The respondents entered their height and body mass in the proprietary survey questionnaire. They were used by the researchers to calculate BMI kg/m2.

(3) Short version of International Physical Activity Questionnaire (IPAQ) was included in it the frequency and time of physical activity with high, moderate, and low intensity, lasting continuously for at least 10 minutes. The percentage of sufficiently active subjects was established on the basis of the estimated caloric costs of physical activity, using the assumptions of Paffenbarger et al.

The results were classified according to the following criteria:

- Low or trace physical activity (below 600 MET · min/week)
- Moderate physical activity (between 600 and 1500 MET · min/week)
- Increased physical activity (1500-3000 MET · min/ week, 1-2 days a week of intensive activity)
- High physical activity (at least 3000 MET · min/ week or above 1500 MET · min/ week but at least 3 days a week with intense activity [10].

Trace or low physical activity (below 600 MET · min/week) justified a description of the sedentary lifestyle [12, 13].

(4) Oswestry Disability Index (ODI) includes 10 questions about everyday activities such as pain severity, self-care, ability to lift weights, walking, sitting, standing, sleeping, social life, traveling, and professional work. Each answer was grated according to the following algorithm: (A) 0 points, (B) 1 point, (C) 2 points, (D) 3 points, (E) 4 points, and (F) 5 points. Then points were summed up. The maximum number of points amounted to 50. This questionnaire was composed of closed questions to choose from (disjunctive question) [12, 14].

The following respondents were excluded from the study: those undergoing treatment due to autoimmune disease/cancer, people with a history of osteoporotic fracture, pregnant women, and respondents who had shown symptoms which could presumably constitute the so-called “red flags” that signal specific low back pain (urinary and bowel incontinence, unexplained weight loss during 3 months before the survey, and serious trauma up to 3 months before the survey).

In the course of the research, a database of variables was created, containing statements, opinions, and evaluations of respondents, enabling the use of computational techniques. Statistical analysis began with substantive and logical control of the collected data. In the first stage, the conformity of the tested continuous variables with the Gaussian distribution was verified. The analysis relied on and univariate logistic regression model and the univariate linear regression model. The t-test was used to verify the significance of the linear regression. This was aimed at eliminating the interfering influence of the various independent variables analysed. The statistical significance level was taken to be p<0.05. The statistical analysis was conducted using STATA/1.C 12.1 software developed by Stata Corp, LP, Texas, USA.

The study was funded by Medical University of Bialystok. In order to conduct the study, approval was obtained from the Bioethics Commission at the Medical University in Bialystok.

## 3. Results

Recurring or chronic low back pain was reported by 355 people (58.29% of all subjects), of which 253 people (41.54% of all respondents) complained of recurrent low back pain.

Respondents with the younger age (under 40 years old) and shorter length of employment (under 15 years) were predominating in the group with recurrent low back pain: 146 people (57.71%) and 167 people (66.01%), respectively.

Chronic low back pain was declared by 102 people (16.75% of all respondents). Total number of 302 people met the criteria of sedentary lifestyle (49.59% of all respondents).

The influence of the age and the length of employment on the presence of recurrent and chronic nsLBP is presented in Table 2. In the univariate logistic regression model, with each successive year of life and length of employment, chances for development of recurrent nsLBP decreased by 4.8% and 4.9%, respectively, while with each successive year of life and length of employment chances for development of chronic nsLBP increased by 7.2% and by 7%, respectively.

The frequency of the sedentary lifestyle, types, and concurring predictive factors of nsLBP in the studied group is presented in Table 3.

**Figure 1:**
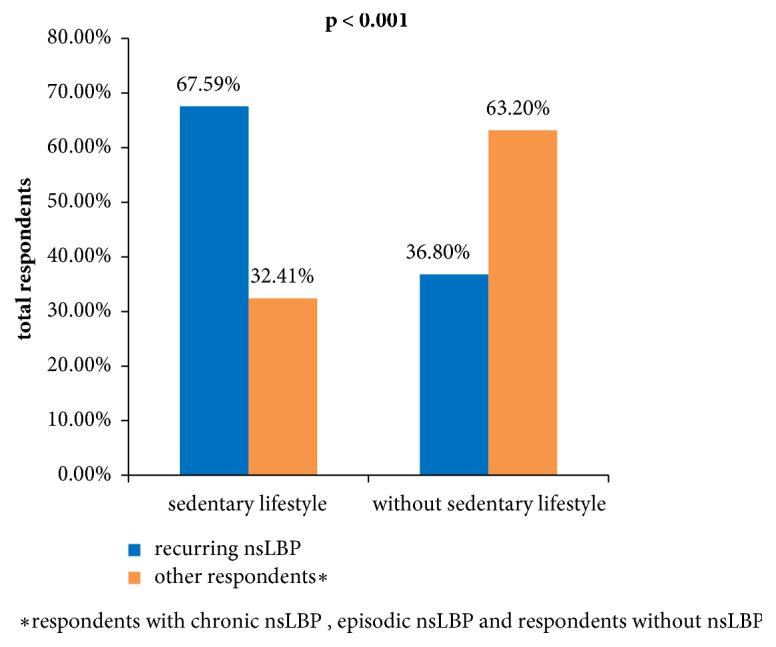
The frequency of recurring low back pain in the groups of respondents, depending on their lifestyle.

Sedentary lifestyle increased the risk of recurrence of nsLBP over 3.5 times (p<0.001) (Table 4, Figure 1.). On the contrary, chances of chronic nsLBP increased over 10 times in people with high physical activity (p<0.001) [Table 4].

The factors associated with lifestyle, such as excessive consumption of coffee (≥ 6 cups per day), increased the risk for nsLBP recurrence by 16 times versus the respondents drinking ≤ 5 cups of coffee a day (OR=16.69; 95% CI: 8.77 – 43.36 (p<0.001), whereas smoking increased the chances of recurrent nsLBP by more than 9 times (OR=9.31; 95% CI: 5.34 -16.22; p <0.001).

In a group of subjects declaring the sedentary lifestyle, the occurrence of recurrent nsLBP was increased by the following components of the metabolic syndrome: hyperlipidemia more than three 3 times, type 2 diabetes more than 3.5 times, and overweight or obesity more than 2 times (p<0.05, p<0.05, p<0.01, respectively). On the other hand, the risk of chronic nsLBP increased by more than 17.5 times in arterial hypertension, more than 4.5 times in diabetes type 2, more than 4.5 times in hyperlipidemia, and more than 4 times in overweight or obesity. The results were statistically significant (p<0.001; p<0.05; p<0.05; p<0.05, respectively) versus the group without these disorders [Table 5].

In the group of respondents with recurrent nsLBP, the sedentary lifestyle was not a factor determining a level of disability, i.e., it did not increase significantly the score based on the Oswestry questionnaire (*β* = 0.236; p=0.756). However, in the respondents with chronic nsLBP, it significantly reduced the score based on Oswestry and reduced the level of disability (*β* = -6.422; p<0.001. In contrast, the comorbidities found in the group of respondents with the sedentary lifestyle with recurrent nsLBP significantly increased the final score by 6.243 points on average for overweight or obesity and 2.415 points on average for hyperlipidaemia (p<0.001; p=0.026, respectively). In the group of respondents with the sedentary lifestyle with chronic nsLBP they significantly increased the final score by 8.168 points on average for overweight or obesity and 4.263 points on average for type 2 diabetes (p<0.001; p=0.003, respectively) [Table 6].

## 4. Discussion

Numerous studies and analyses focused on the excessive strain of the spine and its consequences to health. It was demonstrated that even 2/3 of people below 40 years of age suffered from at least one episode of low back pain and over 60% of them experience a recurrence of pain within the same year [2, 12, 15].

The results of our studies indicate that nonspecific low back pain also represents a significant health problem in the group of professionally active medical personnel, i.e., nurses and paramedics. Over 40% of the respondents complained of recurrent nsLBP, with a significant (p<0.001) prevalence of medical personnel of younger age and length of employment. Martinelli et al. explain this situation with the fact of incorrect use of muscles by the medical personnel during their daily activities at the initial stage of their professional work [16]. The results of our research indicate that, with each year of length of employment, the frequency of recurrent nsLPB decreases significantly (OR=0.95; 95% CI: 0.93-0.97, p<0.001), while the frequency of chronic nsLBP increases significantly (OR = 1.07; 95% CI: 1.03-1.10, p<0.001).

Mannion et al. reached similar conclusions in their prospective study. They report that, for nurses, the frequency of recurrent nsLBP episodes decreased with longer length of employment. They also suggest that this observation may be associated with the fact that medical personnel develops protective adaptation to the increased load [17]. These findings support the appropriateness of preventative programmes dedicated to medical professionals, including educational measures for preventing recurring and chronic low back pain.

The reported data indicate a relationship between nsLBP with the sedentary lifestyle and factors associated with the lifestyle [18, 19]. Our studies demonstrated that the sedentary lifestyle was led by nearly half (49.59%) of all subjects and as many as 67.59% in the group with recurrent nsLBP. In the simple logistic regression model, the sedentary lifestyle increased the chance for development of recurrent nsLBP by over 3.5 times (OR = 3.58; 95% CI: 2.55–5.03, p < 0.001). Hussain et al. in their studies involving 5050 adult Australia inhabitants noted that watching TV for more than 2 hours a day was a predictive factor for low back pain [20]. Similar conclusions were also reached by Falavigna et al. on the basis of their research conducted in a group of medical and physiotherapy students [21].

In our study, the sedentary lifestyle reduced the chances for the development of chronic nsLBP, while a high level of activity significantly increased the chances for the occurrence of recurrent and chronic nsLBP (OR = 1.51; 95% CI: 1.04–2.20 and OR = 10.69; 95% CI: 6.64–17.19, respectively). These results confirm the hypothesis that a relationship between the level of activity and nsLBP can be a U-shaped curve; that is, both inactivity and excessive activity (unhealthy activity) cause an increased risk of back pain [22]. Therefore, this is yet another important conclusion from our study, contradicting a well-spread conviction that the more physical activity the better and indicating a need for appropriate educational activities in this area.

Particularly interesting results of our studies concern the effect of excessive caffeine consumption on recurrent low back pain. Drinking of ≥6 cups of coffee a day increased the chance for the development of recurrent nsLBP by over 16 times versus respondents who did not consume excessive amounts of caffeine (OR=16.69; 95% CI: 8.77–43.36). It should be emphasized that this relationship has not yet been described in the literature. The mechanism underlying that phenomenon has not been fully explained. It is assumed that excessive consumption of coffee may result in flushing magnesium from the body and in lower absorption of calcium, leading to excessive, painful contractions of spine extensor muscles, and lumbar muscles [23].

The results of our study support the thesis that smoking is an important risk factor for recurrent low back pain. In the studied group, smoking increased a chance for recurrent nsLBP by over 9 times (OR=9.31; 95% CI: 5.34–16.22, p < 0.001) versus other people. Studies included in the meta-analysis conducted by Shemory et al. implied that smoking increased a chance for low back pain by over 4 times [11]. Similar conclusions were reached by Karahan et al. for the group of medical personnel and by Smith et al. for nurses [24, 25]. An existence of this relationship was not reported in the research conducted, among others, by Wong et al. [26].

Individual reports are published indicating a relationship between increased total cholesterol serum levels and an increased risk of disc herniation [27]. Suggestions and indirect evidence indicate a certain role played by atherosclerosis in subchondral bone vessels in the pathogenesis of osteoarthritis [28]. In accordance with the results of our studies, hyperlipidaemia increased a chance for development of recurrent and chronic nsLBP by over 3 and 4 times, respectively (OR=3.28; 95% CI: 1.07–10.05 and OR=4.80; 95% CI: 1.21-19.08, respectively).

The results of our study indicate that, in the group with the sedentary lifestyle, concurrent type 2 diabetes increased a chance for recurrent nsLBP by over 3.5 times (OR=3.50; 95% CI: 1.15–10.68) versus people not suffering from diabetes (p=0.027). This observation is consistent with the results obtained by Ha et al. [10]. It is suggested that type 2 diabetes may lead to earlier degeneration of vertebral discs [29].

According to the results of our studies, hypertension significantly increased a chance for development of chronic pain in the lumbosacral spine by over 17.5 times (OR=17.905; 95% CI: 4.43–72.34). This relationship is consistent with the results of very scarce research on this subject [30, 31].

We demonstrated that over half of the respondents with nsLBP were overweight or obese, with a higher percentage found for respondents with chronic nsLBP (59.8%). In respondents declaring the sedentary lifestyle, overweight or obesity significantly increased chances for occurrence of recurrent and chronic nsLBP (OR=2.18; 95% CI: 1.37–3.48 and OR=4.02; 95% CI: 1.08 –14.91; p=0.03, respectively) versus the group without overweight or obesity (p<0.001 and p=0.03, respectively). Results of previous studies conducted by other authors are ambiguous. Croft et al. demonstrated that higher body weight was a predictive factor for pain in the lumbosacral spine in a group of women [32]. Contrary to these results, other researchers [33, 34] did not demonstrate a significant relationship between overweight and obesity and low back pain in a group of nursing personnel.

It is generally thought that low back pain may be a factor determining the occurrence of disability, which, apparently, should result in decreased physical activity. However, a meta-analysis conducted by Lin et al. did not show such a relationship. On the contrary, the researchers demonstrated that people complaining of chronic nsLBP declared a high level of physical activity [35]. Also, our own research did not find a significant relationship between the level of physical activity and the level of disability in subjects with recurrent and chronic nsLBP. Hussain et al. reached opposite conclusions, proving the existence of a relationship between the sedentary lifestyle and the level of disability [20].

The data in the literature indicate that diseases concurrent with nsLBP may be factors determining the level of disability, and accepted predictive factors for disability associated with nsLBP are overweight and obesity [36]. These observations are consistent with the results of our research. Concurrent overweight or obesity significantly increased the final score in the Oswestry questionnaire, i.e., the level of disability in groups of respondents with recurrent and with chronic low back pain (p<0.001; p<0.001, respectively). Also, the presence of hyperlipidaemia or type 2 diabetes in the group of respondents with recurrent and chronic nsLBP significantly determined the level of disability (p<0.02 and p<0.003, respectively). Tsuboi et al., studying 316 healthcare employees with nsLBP, demonstrated a significant relationship between the functional disability and a concurrent metabolic syndrome [37]. However, this relationship requires confirmation in further studies.

## 5. Conclusions

(1) A large proportion (over 40%) of recurring nonspecific low back pain is an important health concern in the nursing and paramedical professions.

(2) A sedentary lifestyle significantly increased the incidence of recurrent low back pain, while increased physical activity had a significant effect on the occurrence of chronic low back pain.

(3) Within the group of individuals leading a sedentary lifestyle, a significant effect of metabolic syndrome components as well as excessive coffee consumption was found on the increased probability of nonspecific low back pain. This finding would support earlier suggestions of recognising these diseases as “novel” predictive factors of nonspecific low back pain.

(4) The results of our study support the appropriateness of preventative programs targeting so-called “diseases of affluence” within medical professionals, including educational measures for preventing recurring and chronic low back pain (with a special emphasis on the importance of recreational physical activity, smoking cessation, maintaining a healthy body mass and instruction in the biomechanical hygiene of the spine).

## Figures and Tables

**Table 1:** General characteristic of the study group.

**Variables**		**N**	%
Profession	Nurses	324	53.20
	Paramedic	285	46.80

Age (years)	30 – 40	302	49.60
	41 – 60	307	50.40

Sex	Women	362	59.94
	Men	247	40.56

	18.5 - 24.99	314	51.56
BMI [kg/m^2^]	25.00 – 29.99/ ≥ 30.00	295	48.44

nsLBP incident in the study group	Recurrent	253	41.54
	Chronic	102	16.75

BMI: body mass index, nsLBP: nonspecific low back pain.

**Table 2:** The influence of the age and the length of employment on the presence of recurrent and chronic nsLBP.

Predictors	Recurrent nsLBP			Chronic nsLBP		
	OR	95 CI	p	OR	95 CI	p
Age [years]	0.952*∗∗*	0.929 – 0.976	<0.001	1.072*∗*	1.037 – 1.168	<0.001

Lenght of employment [years]	0.951*∗∗*	0.928 – 0.974	<0.001	1.070*∗*	1.035 – 1.105	<0.001

OR-odds ratio,

*∗*The risk of nsLBP was significantly increased.

*∗∗* The risk of nsLBP was significantly decreased (the univariate logistic regression model).

**Table 3:** The study population presented in the context of physical activity in the week before filling the questionnaire and concomitant/potential predictive factors of nsLBP.

**Variables**	**Total respondent group (N)**	**Respondents with recurrent nsLBP** **N(**%**)**	**Respondents with chronic nsLBP** **N(**%**)**
Sedentary lifestyle	302	171 (67.59)	13 (12.75)

Moderate physical activity	106	8 (3.16)	17 (16.67)

Increased physical activity	57	3 (1.19)	5 (4.90)

High physical activity	144	71 (28.06)	67 (65.69)

Overweight or obesity	298	140 (55.34)	61 (59.8)

Smoking tobacco	370	214(84.58)	71 (69.61)

Excessive coffee consumption	311	41(16.21)	5 (4.9)

Hyperlipidemia	45	31 (68.89)	14 (13.73)

Arterial hypertension	34	3 (8.82)	21 (20.59)

Diabetes type 2	58	36 (62.07)	20 (19.61)

**Table 4:** Occurrence of non-specific low back pain, depending on the levels of physical activity.

**The level of physical activity**	**Recurrent nsLBP**		**Chronic nsLBP**	
	**OR (95 **%**CI)**	**p**	**OR (95**%** CI)**	**p**
Sedentery lifestyle	3.582 (2.549 – 5.033)*∗*	<0.001	0.110 (0.060 – 0.202)*∗∗*	<0.001
Moderate	0.086 (0.041 – 0.181)*∗∗*	<0.001	0.939 (0.532 – 1.659)	0.829
Increased	0.067 (0.021 – 0.217)*∗∗*	<0.001	0.451 (0.176 – 1.159)	0.098
High	1.512 (1.038 – 2.203)*∗*	0.031	10.690 (6.646 – 17.195)*∗*	<0.001

OR- odds ratio,

*∗*the risk of nsLBP was significantly increased; *∗∗* the risk of nsLBP was significantly decreased (the univariate logistic regression model).

**Table 5:** The influence of the particular components of the metabolic syndrome on prevalence of recurrent nonspecific lower back pain in patients declaring sedentary lifestyle.

**Metabolic syndrome component**	**Recurring nsLBP**		**Chronic nsLBP**	
	**OR** **(95 **%**CI)**	**p**	**OR** **(95 **%**CI)**	**p**
Smoking	9.305 (5.339 – 16.219)*∗*	<0.001	0.821 (0.262 – 2.576)	0.735
Excessive coffee consumption ( ≥ 6 cups for day)	16.688 (8.771 – 43.363)*∗*	<0.001	0.299 (0.038 – 2.343)	0.250
Overweight or obesity	2.182 (1.368 – 3.481)*∗*	0,001	4.020 (1.084 – 14.913)*∗*	0.038
Hiperlipidemia	3.277 (1.069 – 10.050)*∗*	0.038	4.800 (1.207 – 19.082)*∗*	0.026
Diabetes type 2	3.505 (1.150 – 10.680)*∗*	0.027	4.517 (1.141 – 17.875)*∗*	0.032
Arterial hypertension	0.160 (0.034 – 0.756)*∗∗*	0.021	17.905 (4.432 – 72.340)*∗*	<0.001

OR: odds ratio; 95 %CI: confidence interval for OR.

*∗*The risk of nsLBP was significantly increased; *∗∗* the risk of nsLBP was significantly decreased (the univariate logistic regression model).

**Table 6:** The impact of particular types of metabolic syndrome component on higher values on a point scale based on the Oswestry questionnaire in a group of respondents with recurrent or chronic nsLBP declaring sedentary lifestyle.

**Metabolic syndrome component**	**Recurrent nsLBP**				**Chronic nsLBP**			
	**Non- standardized** **beta coefficient**	**SE** **∗** **∗** **∗**	**t**	**p**	**Non- standardized** **beta coefficient**	**SE** **∗** **∗** **∗**	**t**	**p**
Smoking	0.379	0.987	0.384	0.701	1.23	1.264	0.914	0.701

Excessive coffee consumption (≥ 6 cups for day)	-1.502*∗∗*	0.744	-2.018	0.045	1.711	2.699	0.634	0.527

Overweight or obesity	6.243*∗*	0.599	10.425	<0.001	8.168*∗*	0.867	9.424	<0.001

Hyperlipidemia	2.415*∗*	1.076	2.244	0.026	0.479*∗*	1.696	0.282	0.778

Diabetes type 2	0.488	1.020	0.479	0.633	4.263*∗*	1.408	3.029	0.003

Hypertension	-2.893	3.288	-0.880	0.380	-1.489	1.436	-1.036	0.303

SE: standard error.

*∗* The average score obtained on the Oswestry questionnaire was significantly increased, test t.

*∗∗* The average score obtained on the Oswestry questionnaire was significantly decreased, in the univariate linear regression model, test t.

## Data Availability

Data are available any time from the first author, Dr. Anna Citko.
